# Analysis of Readmissions to The Intensive Care Unit After Coronary Artery Bypass Surgery: Ten Years’ Experience

**DOI:** 10.21470/1678-9741-2019-0299

**Published:** 2020

**Authors:** Kamil Cantürk Çakalağaoğlu, Emre Selçuk, Hasan Erdem, Ahmet Elibol, Cengiz Köksal

**Affiliations:** 1 Department of Cardiovascular Surgery, Bakırköy Sadi Konuk Research and Education Hospital, Istanbul, Turkey.; 2 Department of Cardiovascular Surgery, Mus State Hospital, Istanbul, Turkey.; 3 Department of Cardiovascular Surgery, Kartal Kosuyolu Research and Education Hospital, Istanbul, Turkey.; 4 Department of Cardiovascular Surgery, Medical Faculty, Bezmialem Vakif University, Istanbul, Turkey.

**Keywords:** Coronary Artery Bypass, Patient Readmission, Intensive Care Unit, Postoperative Complications, Ventricular Dysfunction, Left, Retrospective Studies

## Abstract

**Objective:**

To evaluate the frequency, causes, and related predictive factors of intensive care unit (ICU) readmissions after coronary artery bypass grafting (CABG) surgery.

**Methods:**

A total of 4112 consecutive patients who underwent on-pump CABG between January 2007 and January 2017 were retrospectively evaluated. The patients were divided into two groups as patients with and without ICU readmission. Demographic and perioperative characteristics were compared between the two groups.

**Results:**

The ICU readmission rate was 3.5%. The most common reasons for ICU readmissions were respiratory (29%) and cardiac (23.4%) complications. The 90-day mortality risk was significantly higher in the readmitted patients than the non-readmitted patients (22.1% and 1.6%, respectively; *P*<0.001; OR=17.6; 95% CI=11.19-28.41). Severe left ventricular dysfunction, chronic obstructive pulmonary disease, end-stage renal disease, emergency CABG, EuroSCORE II > 5%, cross-clamp time > 35 minutes, postoperative respiratory complications, neurological complications, and cardiac complications showed a strong association with ICU readmissions.

**Conclusion:**

ICU readmission after CABG is associated with an increased mortality rate. Evaluation, not only of patients’ comorbidities, but also of intraoperative conditions and postoperative complications, is important to identify patients at risk for ICU readmission.

**Table:** 

Abbreviations, acronyms & symbols	
**ACS**	**= Acute coronary syndrome**
**CABG**	**= Coronary artery bypass grafting**
**CI**	**= Confidence interval**
**COPD**	**= Chronic obstructive pulmonary disease**
**EuroSCORE**	**= European System for Cardiac Operative Risk Evaluation**
**ICU**	**= Intensive care unit**
**IQR**	**= Interquartile range**
**LV**	**= Left ventricular**
**MDRD**	**= Modification of diet in renal disease**
**OR**	**= Odds ratio**
**SD**	**= Standard deviation**
**SPSS**	**= Statistical Package for the Social Sciences**

## INTRODUCTION

Major complications associated with coronary artery bypass grafting (CABG) surgery and overall risk factors associated with mortality are well defined. Similarly, there are widely used risk models for early mortality after cardiac surgery^[1,2]^. Despite advances in surgical techniques and postoperative management strategies, postoperative complications occur in 13 to 38% of patients^[3,4]^. After the first discharge from the intensive care unit (ICU), a number of patients require ICU follow-up again^[5]^. Potential reasons for ICU readmissions are intraoperative deficiencies, clinical conditions lost in the early postoperative period, premature discharge from ICU in initial hospitalization, aggravation of patients’ comorbidities or defined treatment-related complications. ICU applications both extend the length of stay and lead to a significant financial burden^[6,7]^. Most important of all, the short-term mortality rate is higher in these patients than in those who do not need ICU readmission^[8]^. Therefore, it is important to analyze patients who are readmitted to the ICU after the early postoperative period. In this study, we aimed to perform a detailed analysis of ICU readmissions of isolated on-pump CABG patients operated in our clinic for a period of 10 years. We also investigated the preoperative, intraoperative and early postoperative predictive factors of ICU admissions.

## METHODS

All patients who underwent conventional surgical revascularization (on-pump CABG; internal mammary artery to left anterior descending artery, and saphenous vein for the other vessels) by a single surgical team between January 2007 and January 2017 were retrospectively reviewed. Patients who underwent concomitant operations (such as valve surgery, carotid endarterectomy, peripheral vascular surgery), patients who had previously undergone open-heart surgery, patients who underwent off-pump CABG, and those who had intraoperative mortality or index intensive care follow-up were excluded from the study. Patients were divided into two groups: those with and those without ICU readmission. Reasons for readmissions were evaluated. Preoperative, intraoperative and postoperative predictive factors associated with readmissions were investigated. The first 90-day survival rates were compared between the two groups.

Demographic and clinical information of patients were obtained from hospital records. The first 90-day survival data were collected from the national database. The study was conducted in accordance with the Helsinki Declaration after approval by the institutional ethics committee (protocol number: 2019.4/17-194).

The study hospital is a high-level cardiac surgery center with 7/24 rapid response team. For all patients, the decision to operate was made by the consensus of a cardiologist and at least two cardiac surgeons. Indications for ICU readmissions were determined by the responsible cardiac surgeon during working hours, and by the cardiac surgeon on duty outside the working hours together with the consultation of the doctor in charge.

### Study Variables

Demographic characteristics of the patients (age, sex, body mass index), concomitant diseases (diabetes, hypertension, hyperlipidemia, smoking, chronic obstructive pulmonary disease, peripheral vascular disease, neurovascular disease, end-stage renal disease), left ventricular (LV) functions (severe LV dysfunction: ejection fraction < 30%), history of previous acute coronary syndrome (ACS) (last 3 months), operative priority (emergency/elective) and EuroSCORE II levels were evaluated. *Diabetes mellitus* was diagnosed by fasting blood glucose of 126 mg/dL, blood glucose > 200 mg/dL at any time, or history of *diabetes mellitus*, including those treated with diet, oral medications, or insulin. Hypertension was defined as repeated systemic blood pressure measurements exceeding 140/90 mmHg or treatment with antihypertensive drugs for a known diagnosis of hypertension. Hyperlipidemia was defined as a baseline cholesterol level > 200 mg/dL and/or a low-density lipoprotein cholesterol level > 130 mg/dL or previously diagnosed and treated hypercholesterolemia. Current smokers were those who smoked regularly in the past 6 months. Patients diagnosed with chronic obstructive pulmonary disease (COPD) by consultant pulmonologist in the preoperative evaluation were accepted as COPD patients. More than 50% stenosis or total occlusion of one of the carotid arteries; history of amputation due to peripheral arterial disease; presence of a previous or planned intervention to the abdominal aorta, carotid or lower extremity was evaluated as peripheral arterial disease. Patients with renal replacement therapy or with a glomerular filtration rate < 150 ml/min/1.73 m² or renal replacement therapy were considered to have end-stage renal disease. Estimated glomerular filtration rate was calculated for each patient according to the Modification of Diet in Renal Disease (MDRD) equation^[9,10]^. Patients with previous transient ischemic attacks and/or cerebrovascular events were considered to have a history of neurovascular disease.

In-hospital complications were classified as respiratory (pulmonary edema, atelectasis, pneumothorax, exacerbation of COPD, pulmonary embolism, pleural effusion, pneumonia, acute respiratory distress syndrome, aspiration), cardiac (arrest, arrhythmia, low cardiac output syndrome, postoperative myocardial infarction); neurological (major ischemic stroke, transient ischemic attack, cerebral hemorrhage, peripheral neuropathy); gastrointestinal (bleeding, mesenteric ischemia); acute kidney injury (Kidney Disease Improving Global Outcome - KDIGO stage 2-3 or hemodialysis)^[11,12]^; re-exploration (hemorrhage, tamponade), infection. Since the number of patients in need of intensive care more than once was low, the first indication for intensive care readmission was taken based on those patients.

### Statistical Analysis

Categorical variables were presented as counts and frequencies; continuous variables as mean (standard deviation, SD) or median (interquartile range, IQR) as appropriate. Chi-square test or Fisher’s exact test was used for comparison between categorical variables. Student’s t-test or Mann-Whitney U test was used to compare continuous variables. All preoperative characteristics (age > 60 years, BMI > 30 kg/m^2^, male, *diabetes mellitus*, hypertension, hyperlipidemia, current smoking, COPD, peripheral vascular disease, end-stage renal disease, history of ACS, neurovascular disease, emergency CABG, EuroSCORE II > 5%), intraoperative variables (cardiopulmonary bypass time >180 minutes, cross-clamp time > 35 minutes, number of grafts > 3) and postoperative complications were analyzed for ICU readmission. The final model was constructed by independent predictive factors for ICU readmissions by using backward stepwise likelihood ratio logistic regression analysis. The most significant variables that predicted ICU readmission were determined at the last step. Odds ratio (OR) and 95% confidence interval (CI) were reported for all variables. A two-tailed *P*-value of 0.05 was considered statistically significant. SPSS version 23.0 (SPSS, Chicago, IL) was used for all statistical analyzes.

## RESULTS

**Fig. 1:**
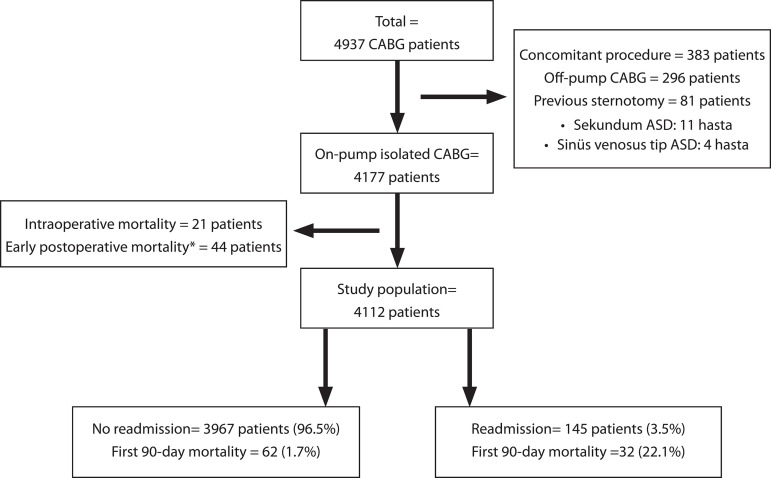
Study population. CABG=Coronary artery bypass grafting. * Patients died during index intensive care follow-up.

A total of 4112 consecutive patients who met the patient selection criteria were evaluated during the study period (Figure 1).

The ICU readmission rate was 3.5% (n=145 patients). Four patients needed intensive care twice, and 3 patients needed intensive care more than twice. The mean age of patients who were readmitted to the ICU was significantly higher than patients without readmission. In patients with ICU readmission, severe left ventricular dysfunction, diabetes, COPD, and end-stage renal disease were much more pronounced compared to the other patients. There was no significant difference between the two groups in terms of the number of bypass grafts. However, in patients who were readmitted to the ICU, the cardiopulmonary bypass and cross-clamp times were longer compared to the others. Table 1 summarizes the clinical characteristics of the two groups of patients.

**Table 1:** Demographic and clinical characteristics of study patients.

		Total	No readmission	Readmission	*P*-value
Preoperative	Patients, n (%)	4112 (100)	3967 (96.5)	145 (3.5)	-
	Age (years), mean (SD)	62.4 (8.2)	59.7 (9.4)	65 (8)	0.002
	BMI (kg/m^2^), mean (SD)	27.8 (4.5)	28.7 (4.5)	28.30 (3.9)	0.606
	Gender, n (%)				
	Male	3142 (76.4)	3023 (76.2)	119 (82.1)	
	Female	970 (23.6)	944 (23.8)	26 (17.9)	
	Severe LV dysfunction, n (%)	183 (4.5)	160 (4)	23 (15.8)	0.001
	*Diabetes mellitus*, n (%)	1192 (29)	1142 (28.8)	50 (34.5)	0.13
	Hypertension, n (%)	2008 (48.8)	1943 (48.9)	65 (44.8)	0.29
	Hyperlipidemia, n (%)	252 (8.6)	1626 (44)	55 (37.9)	0.24
	Current smoking, n (%)	1717 (42.5)	1700 (42.8)	55 (37.9)	0.42
	COPD, n (%)	121 (3.2)	81 (2)	17 (11.7)	< 0.001
	Peripheral vascular disease, n (%)	265 (6.4)	340 (8.6)	12 (8.2)	0.74
	End-stage renal disease, n (%)	139 (3.4)	247 (6.2)	18 (12.4)	0.008
	History of ACS, n (%)	1369 (33.5)	1319 (33.3)	50 (34.5)	0.57
	Neurovascular disease, n (%)	142 (3.5)	125 (3.2)	17 (6.2)	0.07
	Emergency CABG, n (%)	70 (1.7)	61 (1.5)	9 (6.2)	< 0.001
	NYHA class 3-4	974 (23.7)	938 (23.6)	36 (24.8)	0.765
	EuroSCORE II				0.001
	<2%	2863 (69.6)	2806 (70.7)	57 (39.3)	
	2-5%	1033 (25.1)	977 (24.7)	56 (38.6)	
	>5	256 (5.3)	184 (4.6)	32 (22.1)	
Intraoperative	CPB time (min), mean (SD)	81.5 (38.2)	81 (36.5)	93 (41.1)	0.01
	Cross-clamp time (min), mean (SD)	34.1 (15.2)	33.8 (14.5)	38.2 (16.9)	0.002
	Number of grafts, mean (SD)	2.75 (1.7)	2.75 (1.8)	2.82 (0.9)	0.67
Postoperative complications	Pulmonary, n (%)	231 (5.6)	191 (4.8)	40 (27.6)	< 0.001
	Cardiac, n (%)	287 (7.0)	245 (6.2)	42 (29)	< 0.001
	Neurological, n (%)	136 (3.3)	115 (2.9)	21 (14.5)	< 0.001
	Gastrointestinal, n (%)	84 (2.0)	79 (2)	5 (3.4)	0.22
	New-onset dialysis, n (%)	7 (2.4)	91 (2.3)	6 (4.1)	0.14
	Re-exploration, n (%)	179 (4.4)	166 (4.2)	13 (9.0)	0.006
	Infection, n (%)	54 (1.3)	51 (1.3)	3 (2.1)	0.44

ACS=acute coronary syndrome; BMI=body mass index; CABG=coronary artery bypass grafting; CKD=chronic kidney disease; COPD=chronic obstructive pulmonary disease; CPB=cardiopulmonary bypass; LV=left ventricle; NYHA=New York Heart Association

A total of 551 patients (13.4%) faced one of the complications identified in the early postoperative period. Two different complications occurred in 158 (3.8%) patients and 60 (1.4%) patients had more than two complications. The rates of patients needing re-exploration and/or facing respiratory, cardiac and neurological complications in the early postoperative period were significantly higher among those who were readmitted to the ICU than those who were not.

**Fig. 2:**
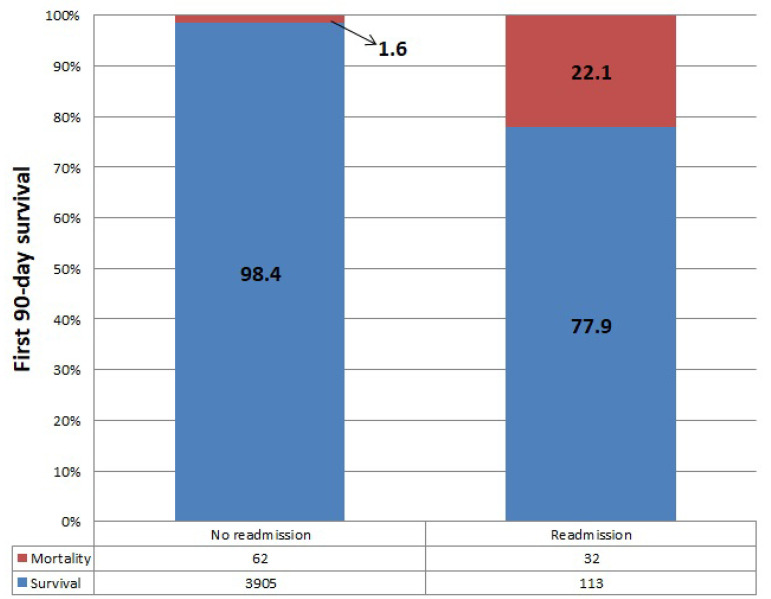
First 90-day survival of study patients.

The etiologies of ICU readmissions were respiratory (29%), cardiac (23.4%), neurological (18.6%), re-exploration (8.3%), renal (8.3%), infection (6.9%), psychiatric (3.4%) and gastrointestinal (2.1%), respectively. Table 2 shows the indications for ICU readmissions. The 90-day mortality rate of the patients included in the study was 2.3% (n=94 patients). The 90-day mortality rate was significantly higher in the ICU readmission group compared to the other patients (22.1% and 1.6%, respectively; *P*<0.001; OR: 17.6; 95% CI: 11.19-28.41) (Figure 2).

**Table 2:** Etiologies of intensive care unit readmissions.

	Readmitted patients	Deaths after ICU readmissions
	n=145	n=32
Pulmonary, n (%)	42 (29)	11 (34.3)
Pulmonary edema*	13 (9)	4 (12.5)
Pneumothorax	2 (1.4)	-
Atelectasis	2 (1.4)	-
Exacerbation of COPD	4 (2.8)	-
Pulmonary embolism	2 (1.4)	1 (3.1)
Pleural effusion	2 (1.4)	-
Pneumonia	8 (5.5)	3 (9.4)
ARDS	6 (4.1)	2 (6.3)
Pulmonary aspiration	3 (2.1)	1 (3.1)
Cardiac, n (%)	34 (23.4)	10 (31.3)
Arrest	9 (6.1)	4 (12.5)
Arrhythmia	14 (9.7)	2 (6.25)
LCOS	8 (5.5)	1 (3.1)
Postoperative MI	3 (2.1)	2 (6.25)
Neurological, n (%)	27 (18.6)	6 (18.75)
Major stroke	8 (5.5)	4 (12.5)
TIA	12 (8.3)	-
Cerebral hemorrhage	6 (4.1)	2 (6.25)
Peripheral neuropathy	1 (0.7)	-
Psychiatric, n (%)	5 (3.4)	-
*Delirium*	4 (2.8)	-
Psychotic episode	1 (0.7)	-
Gastrointestinal, n (%)	3 (2.1)	1 (3.1)
Bleeding	2 (1.4)	-
Mesenteric ischemia	1 (0.7)	1 (3.1)
Renal, n (%)	12 (8.3)	1 (3.1)
AKI (hemodialysis +)	5 (3.4)	1 (2.9)
AKI (hemodialysis -)	7 (4.8)	-
Re-exploration, n (%)	12 (8.3)	1(3.1)
Bleeding	4 (2.8)	-
Tamponade	8 (5.5)	1 (3.1)
Infection, n (%)	10 (6.9)	2 (6.25)
Sternal infection/dehiscence	6 (4.1)	1 (3.1)
Saphenous incision site	3 (2.1)	-
Sepsis	1 (0.7)	1 (3.1)

AKI=acute kidney injury; ARDS=acute respiratory distress syndrome; COPD=chronic obstructive pulmonary disease; ICU=intensive care unit; LCOS=low cardiac output syndrome; MI=myocardial infarction; TIA=transient ischemic attack

* Noncardiogenic

**Fig. 3:**
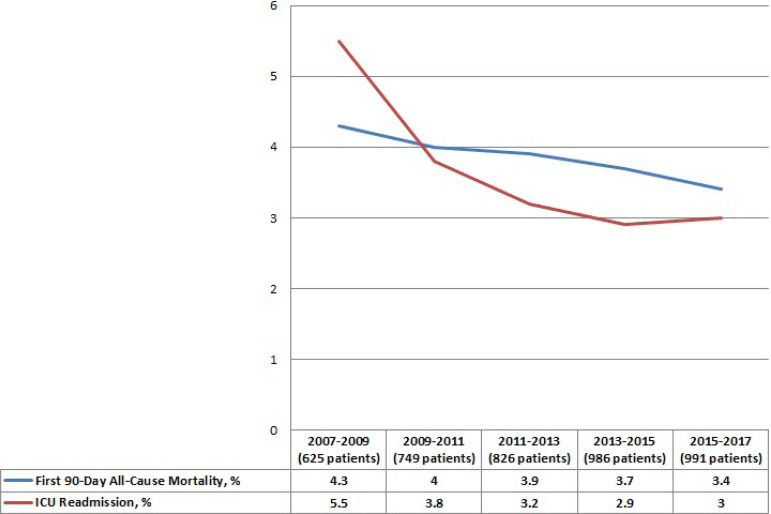
ICU readmission and overall mortality rates during the study years.

The first 90-day mortality rate of all patients who underwent isolated on-pump CABG (n=4177 patients) was similar during the study period but ICU readmission rate showed a significant decrease from 5.5% in 2007 to 3% in 2017, respectively (Figure 3, *P*=0.048)

In multivariate logistic regression analysis, five preoperative (severe left ventricular dysfunction, COPD, end-stage renal disease, emergency CABG, EuroSCORE II > 5%); one intraoperative (cross-clamp time >3 5 minutes) and three postoperative (respiratory, neurological and cardiac complications) independent predictive factors were determined (Table 3, Nagelkerke R^2^=0.545).

**Table 3:** Independent predictive factors of intensive care unit readmissions.

		OR (95% CI)	*P*-value
Preoperative	Ejection fraction <30%	1.72 (1.12-2.6)	0.01
	COPD	4.21 (2.68-7.48)	<0.001
	End-stage renal disease	1.89 (1.02-4.47)	0.02
	Emergency CABG	2.27 (1.28-6.35)	<0.001
	EuroSCORE II >5%	2,71 (1.04-5.32)	0.002
Intraoperative	Cross-clamp time >35 minutes	2.22 (1.42-7.51)	0.03
Postoperative	Pulmonary complications	2.21 (1.26-3.19)	<0.001
	Cardiac complications	3.15 (1.6-4.55)	< 0.001
	Neurologic complications	2.32 (1.51-4.21)	< 0.001

CABG=coronary artery bypass grafting; COPD=chronic obstructive pulmonary disease

## DISCUSSION

In this study, which reflects the single-center experience for ten years, the overall ICU readmission rate is 3.5%. The first 90-day mortality rate was significantly higher in patients with readmission than patients without readmission. Ejection fraction < 30%, chronic obstructive pulmonary disease, end-stage renal disease, emergency CABG, EuroSCORE II > 5%, prolonged cross-clamp time (> 35 minutes), respiratory complications, neurological complications and cardiac complications were independent predictors for ICU recidivism. After adult cardiac surgery, 1.8 to 7.8% of patients need intensive care again for any reason^[13,14]^. This rate varies according to the patient’s profile, type of operation, and center characteristics. The ICU readmission rate after coronary artery bypass surgery was reported to be between 3.1 and 5.3%^[15,16]^. In our study, the ICU readmission rate was 3.5%, which was consistent with the literature.

Respiratory causes have been emphasized in many studies as the most common cause of ICU readmission^[16-20]^. In some series, cardiac causes were more pronounced^[13,21]^. In our study, the most common reason for ICU readmission was respiratory pathologies, followed by cardiac causes. In addition, respiratory and cardiac complications constitute the main causes of mortality in these readmissions.

EuroSCORE II is a universally accepted scoring system for early death after cardiac surgery. In addition, several studies have shown that high EuroSCORE II levels are characterized by an increased ICU readmission risk^[19,22,23]^. In our study, COPD, severe LV dysfunction, emergency and end-stage renal disease, which are also components of the EuroSCORE II system, were significant predictors for ICU readmissions *per se*, independent of EuroSCORE II levels. From this point of view, it seems reasonable to develop more protective strategies in patients with the above-mentioned comorbidities during the postoperative period besides the classical assessment of operative risk.

In our study, although the number of vessels bypassed was similar in both patient groups, prolonged cross-clamp time was an independent risk factor for ICU readmission. Similarly, Benetis et al.^[24]^ reported that patients with long cross-clamp time were associated with more intensive care readmissions. We believe that this situation has two possible causes: first, the prolonged cross-clamp time, as well as systemic side effects, may trigger stubborn complications such as reduced myocardial circulation, postoperative myocardial infarction, low cardiac output and arrhythmia. Another possible reason is that patients with diffuse coronary lesions requiring long cross-clamp processes are more vulnerable to postoperative complications. In addition to preoperative and intraoperative predictive factors, early postoperative complications (mainly cardiac, respiratory and neurological) were strongly associated with the return to the ICU. In this vulnerable patient group, closer follow-up after index ICU stay may reduce the ICU recidivism.

Another important point that must be emphasized in the ICU readmission is its close relationship with early mortality. In our patient group, the 90-day mortality risk was 19.6 times higher in patients with ICU readmission than in other patients. Although van Diepen et al.^[8]^ emphasized that the frequency of admissions after valve surgery is higher than in other patients, in a study by Litwinowicz et al.^[13]^ involving 10,992 patients, the mortality rate was 26% in CABG patients readmitted in the ICU while this rate was 19% in patients undergoing valve surgery. Risk factors associated with atherosclerosis (smoking, hyperlipidemia, diabetes, and hypertension) and frequent comorbidities (such as COPD, neurovascular disease, chronic kidney disease) in CABG patients may contribute to the high mortality rate in ICU readmissions. Another potential cause is not only difficult to manage, but also a high risk of mortality from cardiac complications such as postoperative myocardial infarction in CABG patients^[25]^. In our patient group, mortality was observed in 11 of the 34 patients who were taken to the ICU due to cardiac reasons, and these readmissions were the first in terms of causes of mortality.

The overall first 90-day mortality rate (including intraoperative and early postoperative mortality) has shown a slight decline according to the results of this 10-year experience. Moreover, ICU recidivism was significantly reduced over the years. We believe that these results are associated with improved surgical team experience, improved early postoperative care, and greater effort to identified groups of patients at high risk for ICU readmission.

Current reports highlight the progressive increase in comorbidities of CABG patients over the years^[4,26]^. Therefore, ICU readmissions will continue to remain important as a part of quality assessment after cardiac surgery. Restricted ICU bed capacity compared to the increasing number of patients may adversely affect patient circulation. According to the results of our study, several perioperative factors are associated with a higher risk of ICU readmission. The generation of risk models based on multicenter ICU readmission studies may be useful for optimal care of high-risk patients and appropriate resource utilization.

### Limitations

This large single-center study has well-defined limitations due to the retrospective design of the study. First, this study was conducted in a heterogeneous group of patients over a long period. Naturally, surgeons’ experience, general evolution of the study center and changes in patient profile were reflected in the results of the study. Patient selection criteria were restricted to minimize these effects. The major challenge in the analysis of ICU readmissions was the fact that, for a certain number of patients, pre-diagnosis and the underlying pathologies might have been different. Nevertheless, systematically recorded patient progress reports were useful in identifying complications. Of note, the cardiovascular system has a close relationship with many other systems (such as pulmonary, renal, etc.); therefore, complications from cardiac surgery tend to interact with each other. Especially in the analysis of these complicated ICU readmissions, prospective data extraction process would be particularly reliable.

## CONCLUSION

In this observational study, including 4112 patients who underwent on-pump CABG, the ICU readmission rate was 3.5%. In the last decade, a significant decrease in the ICU readmission rate has been observed. Several perioperative variables were associated with ICU recidivism. To reduce ICU admission rates and improve CABG outcomes, in addition to patient comorbidities, intraoperative and postoperative variables should be considered.

**Table:** 

Authors' roles & responsibilities	
KCC	Substantial contributions to the conception or design of the work; or the acquisition, analysis, or interpretation of data for the work; drafting the work or revising it critically for important intellectual content; agreement to be accountable for all aspects of the work in ensuring that questions related to the accuracy or integrity of any part of the work are appropriately investigated and resolved; final approval of the version to be published
ES	Substantial contributions to the conception or design of the work; or the acquisition, analysis, or interpretation of data for the work; drafting the work or revising it critically for important intellectual content; agreement to be accountable for all aspects of the work in ensuring that questions related to the accuracy or integrity of any part of the work are appropriately investigated and resolved; final approval of the version to be published
HE	Substantial contributions to the conception or design of the work; or the acquisition, analysis, or interpretation of data for the work; drafting the work or revising it critically for important intellectual content; agreement to be accountable for all aspects of the work in ensuring that questions related to the accuracy or integrity of any part of the work are appropriately investigated and resolved; final approval of the version to be published
AE	Substantial contributions to the conception or design of the work; or the acquisition, analysis, or interpretation of data for the work; drafting the work or revising it critically for important intellectual content; agreement to be accountable for all aspects of the work in ensuring that questions related to the accuracy or integrity of any part of the work are appropriately investigated and resolved; final approval of the version to be published
CK	Drafting the work or revising it critically for important intellectual content; agreement to be accountable for all aspects of the work in ensuring that questions related to the accuracy or integrity of any part of the work are appropriately investigated and resolved; final approval of the version to be published
